# Severe dengue: Are type I interferon autoantibodies involved?

**DOI:** 10.70962/jhi.20260214

**Published:** 2026-11-12

**Authors:** Vu Le-Huy Tran, Alessandro Borghesi, Jean-Laurent Casanova, Shen-Ying Zhang

**Affiliations:** 1 Laboratory of Human Genetics of Infectious Diseases, University of Texas Southwestern, Dallas, TX, USA; 2 Center for the Genetics of Host Defense, University of Texas Southwestern Medical Center, Dallas, TX, USA; 3 Neonatal and Pediatric Intensive Care Unit, Mother, Child and Adolescent Department, Geneva University Hospitals, Geneva, Switzerland; 4 Department of Pediatrics, Gynecology and Obstetrics, University of Geneva, Geneva, Switzerland; 5 Host-Pathogen Interaction Unit, San Matteo Research Hospital (Fondazione IRCCS Policlinico San Matteo), Pavia, Italy; 6 Global Health Institute, School of Life Sciences, École Polytechnique Fédérale de Lausanne, Lausanne, Switzerland; 7 Laboratory of Human Genetics of Infectious Diseases, Université Paris Cité, Institut Imagine, Necker Branch, INSERM UMR 1163, Paris, France; 8 Department of Pediatrics, University of Texas Southwestern, Dallas, TX, USA; 9 Department of Immunology, University of Texas Southwestern, Dallas, TX, USA; 10 Children’s Hospital Research Institute, Dallas, TX, USA; 11 Howard Hughes Medical Institute, New York, NY, USA; 12 Department of Neurology, University of Texas Southwestern, Dallas, TX, USA; 13 Department of Neuroscience, University of Texas Southwestern, Dallas, TX, USA

## Abstract

Dengue virus (DENV) is the most rapidly disseminating arbovirus worldwide. A new study by Yang et al. (https://doi.org/10.70962/jhi.20260072) shows that autoantibodies neutralizing type I IFNs (AAN-I-IFN) may underlie severe dengue in ∼14% (7/49) of cases in a cohort of Chinese patients. Further studies of children and adults of different ancestries with silent, benign, and severe DENV infection are warranted.

## Teaser

Dengue virus is the most rapidly disseminating arbovirus worldwide. A new study by Yang et al. shows that autoantibodies neutralizing type I IFNs may underlie severe dengue in ∼14% (7/49) of cases in a cohort of Chinese patients.

Dengue virus (DENV) is the most rapidly disseminating arthropod-borne virus (arbovirus). The earliest documented outbreaks of dengue fever date back to 1771 in Puerto Rico and 1801 in Spain, with multiple suspected dengue outbreaks recorded across the Americas and Asia thereafter. The four serotypes of DENV, DENV-1–4, are genetically related (∼65–70% amino acid sequence identity) but have serotype-specific antigenic epitopes in envelope proteins in addition to conserved, cross-reactive sites, although an antigenic cartography study has suggested that these differences form a continuum rather than discrete clusters (1). All four recognized serotypes are transmitted by *Aedes aegypti* and *Aedes albopictus* mosquitoes. Humans are the main reservoir, but these viruses also infect nonhuman primates and possibly other animals, based on limited serological evidence, during sylvatic/enzootic cycles of dengue. Increasing urbanization, global travel and trade, population growth, and global warming have all facilitated the spread of both the *Aedes* vectors and DENV, increasing the frequency of major dengue outbreaks across the Americas, Africa, Europe, and Southeast Asia in recent decades.

An estimated 50–80% of DENV-infected individuals are asymptomatic and ∼20–50% experience “dengue fever,” a mild-to-moderate acute clinical illness characterized by fever and body aches accompanied by mild hematological and biochemical abnormalities (1). About 10% of symptomatic dengue patients develop warning signs indicating an increase in the risk of progression to life-threatening disease (“severe dengue”), which eventually occurs in only a minority of symptomatic patients (<1–5%) (1). Hemorrhagic manifestations (dengue hemorrhagic fever [DHF]) occur in ∼44% of patients with severe dengue, and plasma leakage leading to dengue shock syndrome (DSS) occurs in ∼22%. Various types of organ complication are observed in patients with severe dengue, including acute liver failure in 17.3% (95% confidence interval [CI] = 6.5–31.5%), cardiac involvement (i.e., cardiac dysfunction, myocarditis) in ∼40–73%, and neurological complications in ∼0.5–∼6% of cases. DENV infection has a relatively low overall case-fatality rate (∼0.05% [2]) and an even lower infection-fatality rate (probably <0.01%), but the large number of cases globally nevertheless translates into a significant number of deaths. In 2025 alone, about 1.7 million confirmed dengue fever cases (almost 4.5 million if suspected cases are also included) were reported in the Americas, with more than 2,200 deaths, mostly in Brazil, Colombia, Ecuador, Peru, and Panama (2).

The severity of DENV infection varies with serotype and is partly dependent on whether the patient has a history of prior infection with a different serotype. Severe dengue is more frequent in patients infected with DENV-2 than in patients infected with the other serotypes, and secondary DENV-2 infection is typically more severe following a primary DENV-1 infection. Clinical severity tends to manifest differently in different age groups, with young children and the elderly experiencing the most severe outcomes, particularly if they have other comorbid conditions (3). The overall mortality of DENV infection follows a roughly J-shaped curve, with moderately high numbers of deaths in infants under the age of 1 year and most deaths affecting elderly individuals over the age of 70 years (3). For secondary DENV-2 infection, mortality follows an asymmetric U-shaped curve skewed toward younger individuals, with death rates higher in children aged 3–14 years than in other age groups. In this context, the cause and mechanism of severe dengue remain unclear.

Since 1996, the study of monogenic inborn errors of immunity (IEI) has clarified the pathogenesis of many life-threatening infectious diseases, including viral diseases. Remarkably, autoantibodies (auto-Abs) neutralizing specific cytokines have been shown to underlie the same severe infections as the corresponding IEI. The most striking example is the discovery in 2020 that auto-Abs neutralizing type I IFNs (AAN-I-IFN), mimicking IEI of IFN-I immunity, underlie about 15% of cases of critical Coronavirus Disease 2019 (COVID-19) pneumonia (4). In this issue of the *Journal of Human Immunity*, Yang et al. report the detection of AAN-I-IFN in ∼14% of cases of severe dengue in a cohort of Chinese patients (5). The authors studied 216 patients (male [M]/female [F] 123/93, aged 50.75 [32.97–61.61] years) infected exclusively with DENV-1 in Guangzhou, China, including seven patients diagnosed with DSS (M/F 4/3, aged 67.00 [52.64–78.35] years, 1/7 with documented secondary infection) and 42 with DHF (M/F 17/25, aged 61.72 [52.02–70.88] years, 2/42 with documented secondary infection). Cardiac injury was reported in five DSS and 15 DHF patients, and cerebral injury was found in three DSS and six DHF patients. Using a cell-based neutralization assay, the authors showed that one in seven DSS (14.3%, 95% CI = 2.6–51.3) and six in 42 DHF patients (14.3%, 95% CI = 6.7–27.9) tested had auto-Abs that neutralized 100 pg/ml IFN-α2 and/or IFN-ω, whereas none of the 167 patients with moderate dengue fever symptoms (all with primary DENV infection) carried these auto-Abs.

All seven IFN-I auto-Ab–positive patients experienced plasma leakage and cardiac injury, but not cerebral injury. Plasma samples from six of these seven patients, tested in an in vitro assay, suppressed the antiviral activity of IFN-α, allowing DENV to replicate rapidly in a cultured cell system, providing evidence of the relevance of these auto-Abs for severe DENV infection. An association was found between these auto-Abs and the risk of myocardial injury, which was observed in all seven auto-Ab–positive patients and in 4/6 (67%) and 8/36 (22%) of the auto-Ab–negative patients in the DSS and DHF disease groups, respectively. After adjustment for age, sex, and comorbid conditions, harboring AAN-I-IFN resulted in ∼23 times higher odds of having cardiac injury (odds ratio [OR] = 22.8, 95% CI = 2.4–3,057.7, P = 0.003) and ∼53 times higher odds of having hypoproteinemia (a marker of plasma leakage; OR = 53.5, 95% CI = 3.6–8,809.2, P = 0.001). A longitudinal analysis of selected samples also suggested that platelet recovery occurred later in DHF patients with IFN-I auto-Abs than in those without these auto-Abs. This report, thus, suggests that AAN-I-IFN may drive severe dengue in adults, adding this illness to the growing list of viral conditions associated with AAN-I-IFN (4).

Since 2020, following the report that these AAN-I-IFN underlie ∼15% of cases of critical COVID-19 pneumonia (4), a number of recent studies have demonstrated the pathogenic role of these auto-Abs in a wide range of severe diseases affecting various organs of the human body following infection with various rare or common, epidemic or emerging viruses (6), including, in particular, a growing number of arboviruses (Table 1). AAN-I-IFN have also been shown to underlie 10%, 35%, or 40% of Tick-borne (7), Chikungunya virus (CHIKV) (8), or West Nile virus encephalitis (9) and 30% or 100% of severe adverse reactions to live-attenuated Yellow Fever virus 17D vaccine (10) or CHIKV vaccine encephalitis (11), respectively. They have also been shown to underlie most cases of severe infection with the rarer Powassan, Usutu, and Ross River viruses (12). Interestingly, one AAN-I-IFN–positive patient with severe Usutu virus infection developed myocarditis (12) resembling the cardiac injury observed in patients with severe dengue and AAN-I-IFN, but further studies are required to confirm such associations. The screening of blood samples from >34,000 healthy French donors showed that AAN-I-IFN were present in the general population, with a prevalence of 0.17% in individuals under 70 years of age and 4% in those over 70 years of age (13), corresponding to an estimated 100 million humans worldwide. Consistent with these findings, a longitudinal HIV cohort analysis revealed that ∼1.9% of individuals over the age of 65 years had IFN-I auto-Abs, first detected at a median age of ∼63 years (14). These IFN-I auto-Abs and the autoreactive B cells that produce them are stable and present before the occurrence of severe viral infections; AAN-I-IFN are the cause rather than a consequence of severe viral diseases (15, 16). The range of viral diseases in individuals with AAN-I-IFN may be even greater than currently thought.

**Table 1.** AAN-I-IFNs underlie a range of arthropod-borne virus (arbovirus) diseases in humans

Arbovirus (Family)	Vector	Arboviral diseases in the cohort studied	AAN-I-IFN^+^ cases/cases tested (%)	Reference
**Naturally occurring arboviral infections**	​	​	​	​
West Nile virus (Flaviviridae)	Mosquitoes	WNV meningitis and encephalitis	206/548 (38%)	(12)
Tick-borne Encephalitis virus (Flaviviridae)	Ticks	TBEV encephalitis	9/89 (10.1%)	(9)
Powassan virus (Flaviviridae)	Ticks	POWV encephalitis	1/1 (100%)	(11)
Usutu virus (Flaviviridae)	Mosquitoes	USUV meningitis, encephalitis, and myocarditis	2/3 (66.7%)	(11)
Ross River virus (Togaviridae)	Mosquitoes	Severe RRV disease	1/24 (4.2%)	(11)
Chikungunya virus (Togaviridae)	Mosquitoes	CHIKV myelitis, encephalitis, and encephalomyelitis	7/20 (35%)	(7)
Dengue virus (Flaviviridae)	Mosquitoes	Dengue hemorrhagic fever and Dengue shock syndrome	7/49 (14.3%)	(5)

Arbovirus (Family)	Vaccine	Postvaccination diseases	AAN-I-IFN^+^ cases/cases tested	Reference
**Severe reactions to live-attenuated arboviral vaccines**	​	​	​	​
Yellow Fever virus (Flaviviridae)	17D	Meningoencephalitis, hepatitis, renal insufficiency, and shock	3/8 (37.5%)	(8)
Chikungunya virus (Togaviridae)	VLA1553	Encephalitis	3/3 encephalitis (100%) or 3/5 severe cases (60%)	(10)

Some caution is required, as this new report concerns a single cohort study of limited sample size, but the results reported by Yang et al. nevertheless suggest that AAN-I-IFN play a pathogenic role in severe DENV infection related to clinical signs and biological markers of cardiac injury and potentially also hypoproteinemia, the hallmarks of severe DENV disease. Intriguingly, this association was observed for both cardiac injury and hypoproteinemia in all AAN-I-IFN–positive patients, whether they had DSS or DHF, rather than being confined to DSS, which is generally more severe clinically. Given that patients with DSS and DHF require close monitoring in hospital, it seems reasonable to assume that auto-Ab–positive patients are at high risk of progressing to critical DENV disease in the absence of appropriate treatment. The study reported here had a limited sample size, with only seven DSS patients and 42 DHF patients tested. Additional studies on a larger cohort will be required to confirm its findings. Moreover, it will be very important to perform similar studies in other cohorts of patients with severe DENV infection from different populations in different countries and in cohorts including children, along with age-matched asymptomatic DENV-infected controls and cases with mild-to-moderate clinical symptoms. This report should trigger new research interest in understanding DENV disease pathogenesis.
